# Plant Bioinspired Ecological Robotics

**DOI:** 10.3389/frobt.2020.00079

**Published:** 2020-07-14

**Authors:** P. Adrian Frazier, Lorenzo Jamone, Kaspar Althoefer, Paco Calvo

**Affiliations:** ^1^MINTLab - Minimal Intelligence Lab, Universidad de Murcia, Murcia, Spain; ^2^Center for the Ecological Study of Perception and Action University of Connecticut, Storrs, CT, United States; ^3^Centre for Advanced Robotics @ Queen Mary (ARQ), School of Electronic Engineering and Computer Science, Queen Mary University of London, London, United Kingdom

**Keywords:** bioinspired robotics, ecological psychology, plant signaling and behavior, endogenous control, tau theory

## Abstract

Plants are movers, but the nature of their movement differs dramatically from that of creatures that move their whole body from point A to point B. Plants grow to where they are going. Bio-inspired robotics sometimes emulates plants' growth-based movement; but growing is part of a broader system of movement guidance and control. We argue that ecological psychology's conception of “information” and “control” can simultaneously make sense of what it means for a plant to navigate its environment and provide a control scheme for the design of ecological plant-inspired robotics. In this effort, we will outline several control laws and give special consideration to the class of control laws identified by tau theory, such as time to contact.

## Introduction

Bioinspired robotics and artificial intelligence has taken various forms, including genetic algorithms, artificial life, and evolutionary robotics (Langton, 1986; Mitchell, 1996; Doncieux et al., 2015); behavior-based and situated robotics (Steels et al., 1995; Arkin, 1998); swarm robotics (Romanishin et al., 2013); morphological computation and soft robotics (Paul, 2006; Pfeifer et al., 2014; Laschi et al., 2016), and others (Calvo and Gomila, 2008). Plants have inspired advances in the material sciences (Mazzolai et al., 2010; Szyndler et al., 2013; Guo et al., 2015; Lucarotti et al., 2015; Voigt et al., 2015) and novel forms of movement based on regeneration, accretion, and eversion (Sadeghi et al., 2014, 2017; Sadeghi et al., 2014, Sadeghi et al., 2017, Greer et al., 2019 Putzu et al., 2018). Elaborate “plantoid” robots come equipped with tree-like branches, leaves, and sensorized, bendable roots, which emulate to some degree the distributed foraging exhibited by plants (Sadeghi et al., 2017). Others have taken inspiration from plant nanoparticles and adhesives (Burris et al., 2018).

Much plant-inspiration remains to be discovered (Vidoni et al., 2015; Wahby et al., 2018) beyond “copying innovations” (Burris et al., 2018). For one thing, innovations along the aforementioned lines resort to pulling out the same bag of tricks that animal researchers have exploited in the past (e.g., materials, morphologies, adhesive nanoproperties, and other biochemical mechanisms), if only rehearsed with plants rather than animal models. But plant bio-inspiration doesn't reduce to transferring biomimetic successes from the animal to the plant kingdom, either in systems and synthetic biology or in molecular and cell biology. Put bluntly, it is not the synthetic gadgets themselves that we are after here. It is rather the grasping and climbing behaviors, the way in which the approaching maneuvers may be controlled, and not their attachment mechanisms or their smart biomimesis.

In what follows, we highlight a role for ecological psychology in both plant science and plant-based robotics. In particular, we explain how successful movement requires an organism (and a robot) to be informed by the very environment as to where to go about. A plant-inspired ecological robot is a robot that can tune to the structure that the surrounding energy media provides. To do so, we suggest, robots could be engineered to exploit the same type of control laws that plants exploit. It is our hope that a plant-inspired ecological robotics will allow researchers to pay due consideration to some new challenges, and opportunities, for robotics and artificial intelligence.

## Plant Movement

As with animals, plants *move* (Darwin and Darwin, 1880; Mugnai et al., 2007; Riviere et al., 2017). They do so to access information about their environments and engage in adaptive interactions with them (Isnard and Silk, 2009; Carello et al., 2012; Gianoli, 2015). But as rooted creatures, plants cannot get up and flee when threatened, if needed (Trewavas, 2017; Calvo et al., 2020). Their survival strategies can in fact vary dramatically. Extremophytes, such as *Anastatica hierochuntica* (the Rose of Jericho, a somewhat distant relative of the Brassicaceae family of the plant model *Arabidopsis thaliana*) exhibits a high degree of metabolism-based tolerance to extreme heat, lack of Nitrogen, or to a salty environment (Eshel et al., 2017). But not all strategies reduce to evolving a resilient metabolism. Plants, for instance, can survive by extending themselves over as much terrain and in as many directions as they can gain access to Gianoli et al. (2012). If a line of growth gets cut off, enough redundancy exists to compensate (Trewavas, 2014). But the foregoing is unlikely to succeed if it proceeds at random. Slow movement time and irreversibility means that the plant can ill-afford to rely on chance alone. Through action, plants are able to sample the environment and tune to information with an adaptive value (Calvo and Friston, 2017). This is especially true of climbing plants (Darwin, 1875), like *Phaseolus vulgaris* (Millet et al., 1988; Badot et al., 1990; Millet and Badot, 1996), the so-called “common bean,” which take big risks by extending themselves upward with little in the way of a supportive trunk. They grow tendrils that sway and whip around in ovular cycles until grabbing hold of something (Caré et al., 1998), providing support for continued upward expansion. While the temptation exists to imagine this process in terms of random ballistic projections, there is reason to believe that such is not the case (Calvo et al., 2017a), nor should it be.

To be successful, plant movement must be informed about where to go by the environment (Carello et al., 2012). The plant control system, like that of any organism, is sensitive to an array of different biotic and abiotic energy media and their structuring, ranging from electromagnetic fields to chemical diffusion gradients, vibrations in air and water, and deformations of its own proprioceptive surfaces (Balusška et al., 2006; Brenner et al., 2006; Bastien et al., 2013, 2015; Dumais, 2013; Calvo et al., 2016; Choi et al., 2016; Gilroy et al., 2016; Huber and Bauerle, 2016). If a plant needs a climbable surface, and if a nearby climbable rod structures ambient light in a way that is *specific* to its climbability, then the plant does not have to guess about where to go (Gibson, 1966, 1979; Carello et al., 2012; Turvey, 2019). Climbability, in this case, would be a function of plant-rod properties, such as distance between plant and rod, stalk-strength, tendril length, curl-tightness, and so on. Assuming all this to be the case, a control law would exist relating the plant's getting-to-climbable-surface relevant activities to the light and its getting-to-climbable-surface-relevant structure. If the plant can tune its activities to that structure, then it can capitalize on it and extend its capacity to exploit the sunlight. Whatever the structure in whatever informational media are involved in climbing and nutation, exploiting control laws has numerous advantages over a blind trial and error. For instance, a control law specifying the rate of approach to the rod would allow the plant to manage inertial forces and avoid bouncing off the rod (Lee, 1998). For the plant-inspired roboticist, this also means offloading control to the environment. As an analogy, consider a six-legged robot moving through a field of debris. If the legs are springy, as with RHex (Altendorfer et al., 2001), then the robot can bounce its way from one side to the other. The debris itself, in its interactions with the legs, will cause the bouncing, all without any computing or explicit instructions. Similar morphological-dynamical coupling can be found with, for instance, bio-tensegrity (Turvey and Fonseca, 2014) and preflexes (Dickinson et al., 2000). Control laws go deeper than purely morphological-dynamical coupling and offer the creature and robot alike the opportunity to *act in advance of acting*.

## Ecological Psychology

Ecological psychology (Gibson, 1966, 1979) was meant to contrast with cognitive psychology in that the emphasis of analysis is the organism-environment relationship, rather than the organism's thoughts. Several more recent theories centering embodiment, embeddedness/situatedness, and enaction have adopted a similar stance (Richardson et al., 2008; Froese and Di Paolo, 2011). Increasingly, these perspectives are making their way into robotics, given that robots are bodied and need to get around in cluttered environments and realize goals (Duchon et al., 1998; Jamone et al., 2016; Zech et al., 2017). For ecological psychologists, the what-is-perceived is not a category of thing, like “rod,” but a climbable surface. In this case, “climbable” is an affordance and “climbing” is an effectivity. Before questions of control can be answered, the roboticist needs to consider whether its robot can do the task at hand. Is the robot a climber? Can it climb? And is climbing available to do? Less trivial is determining if the affordance is specified in the lawfully structured energies to which it is sensitive. An oceanic protist that feeds on photosynthetic bacteria needs to get to the ocean surface. How does it know where to go? If it has a light sensitive pigment coupled to its swimming apparatus, then it should swim so as to increase stimulation of the pigment, because sunlight forms light gradients near the ocean surface (Swenson and Turvey, 1991). The light gradient specifies that food is available (because it is day) and where to go to get it. It is “information about” the location of photosynthetic bacteria. The relationship between swimming and the light gradient is, as previously mentioned, a control law.

### Information and Control Laws

The key principle behind ecological psychology's conception of “specifying (or lawful) information” is that the various ambient energy media surrounding an organism are structured by the dynamics of its environment. At a given point of observation (PO), a set of relations exists between the PO and the field, as well as between the sub-fields defined by local distributions of ambient energy. As either the location of a PO or that of an object in the environment is transformed, so too is the set of relations. An organism differs from a generic PO in that it can only access so much of the field—it has a perspective. Nevertheless, the story is similar to that of the PO. As the organism's perspective (or position of an object in the environment) changes, so too do relations within the subfield. Some relations change systematically, while others remain constant (X changes in size relative to Y, but X is always above Y, relative to the direction of gravity). Most importantly, a relationship between transformations of an organism's perspective are directly related to transformations of the relations between distributions of energy in the sub-field (or, respectively, the PO and the field). The latter constitute “lawful information” about the former. Processes that generate transformations of the organism's perspective constitute the “control structure.” Mathematically, control and information are duals, akin to the relationship between points and lines, where two points define a line, and two intersecting lines define a point (Shaw and Turvey, 1981). Control is governed by transformations of the relations between energy distributions in the sub-field, i.e., information. And transformations of the information present in the sub-field are determined by transformations of perspective, i.e., control. When this is the case, a control law exists. The ability for an organism (or robot) to attune its activities to these laws is what makes transformations of energy informative.

#### The Outfielder Problem

**Figure 1:**
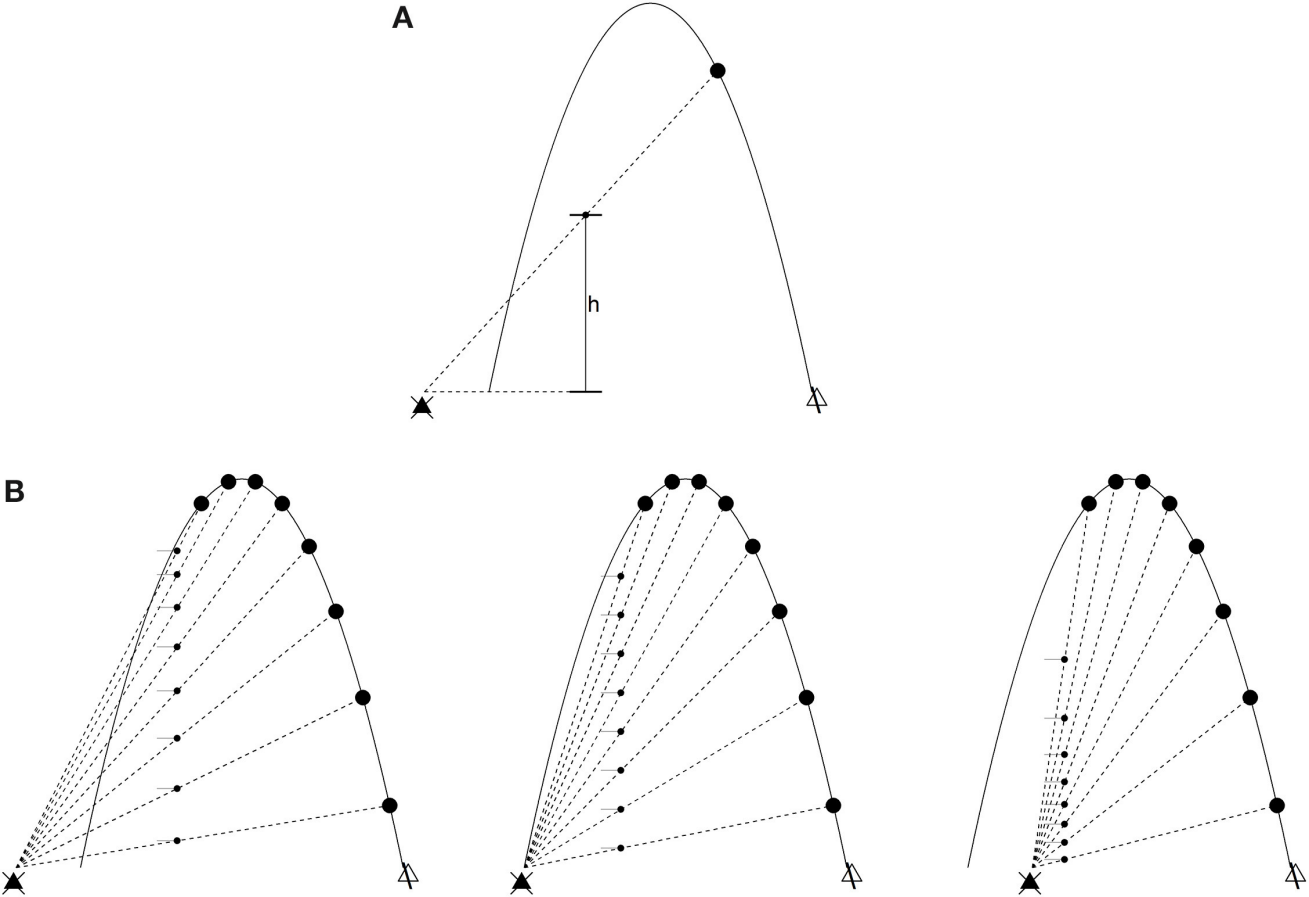
The outfielder problem. The ball (in low-friction conditions) flies in a parabolic trajectory. The large dots are time slices of such a trajectory, and the black triangle on the left is the point of observation (the outfielder's line of sight). **(A)** The optical variable h is the distance between the outfielder's line of sight and a projection of the ball at an arbitrary position x. Optical acceleration is ḧ, the second derivative of h. **(B)** Left: decreasing optical acceleration. The ball will land in front of the outfielder if they do not run forward. Middle: zero optical acceleration. The outfielder will intercept the ball's trajectory. Right: increasing optical acceleration. The ball will land behind the outfielder.

The Chapman strategy (Chapman, 1968) for catching a flyball illustrates the foregoing. Think of an imaginary screen placed at some distance from an outfielder as the batter hits a flyball in their direction. The projection *h*(*t*) of the ball on the screen at a given time *t* is

$$\begin{array}{l} h=\frac{Y{\left(t\right)}_{ball}-Y{\left(t\right)}_{po}}{X{\left(t\right)}_{ball}-X{\left(t\right)}_{po}}, \end{array}$$

where (*X*_*ball*_, *Y*_*ball*_) and (*X*_*po*_, *Y*_*po*_) are the position of the ball and outfielder (respectively) in an inertial coordinate system (see Figure 1A). The *optical acceleration* (OA) is ḧ, the second derivative. Control laws, in this situation, arise from the following (see Figure 1B):

1. If ḧ = 0, the ball will intercept the PO.
2. If ḧ > 0, the ball will fly over the PO.
3. If ḧ < 0, the ball will land in front of the PO.

This means different things depending on whether the outfielder is moving or stationary. If the latter, then the outfielder (1) is positioned to catch the ball, (2) needs to run backwards, or (3) needs to run forwards. If moving at constant velocity in the same direction as the ball, the outfielder (1) is running at the right speed to catch the ball, (2) needs to run at a faster constant velocity, and (3) needs to run at a slower constant velocity. In both cases, the strategy is cancellation of the optical acceleration (Michaels and Oudejans, 1992; McLeod and Dienes, 1993; Rozendaal and van Soest, 2003). The following dynamical law describes this scenario when ḧ = 0 throughout the ball's trajectory (Rozendaal and van Soest, 2003):

$$\begin{array}{l} {Ẍ}_{po}={Ẍ}_{ball}-\frac{X_{ball,po}}{Y_{ball}}{\ddot{Y}}_{ball}-2{Ẋ}_{ball,po}\left(\frac{{Ẋ}_{ball,po}}{X_{ball,po}}-\frac{{Ẏ}_{ball}}{Y_{ball}}\;\right). \end{array}$$

The behavior of the PO under this dynamical law, generally, is initially high acceleration settling into constant velocity, which is consistent with the empirically observed tendency to do the same near interception. The stationary case described earlier suggests a somewhat different strategy, which is to accelerate not at all, backwards, or forwards (respectively). The same strategy can be applied more generally, where constant velocity (near interception of the ball) of the PO is no longer assumed, so that Ẍ_*po*_ = −κḧ. The gain κ is positive under circumstances where both times the ball crosses the outfielder's line of site, it is out in front of the outfielder. This is reversed if the outfielder faces the opposite direction (among other cases; see Rozendaal and van Soest, 2003, for details).

### Optical Flow Fields and the Optical Push

**Figure 2:**
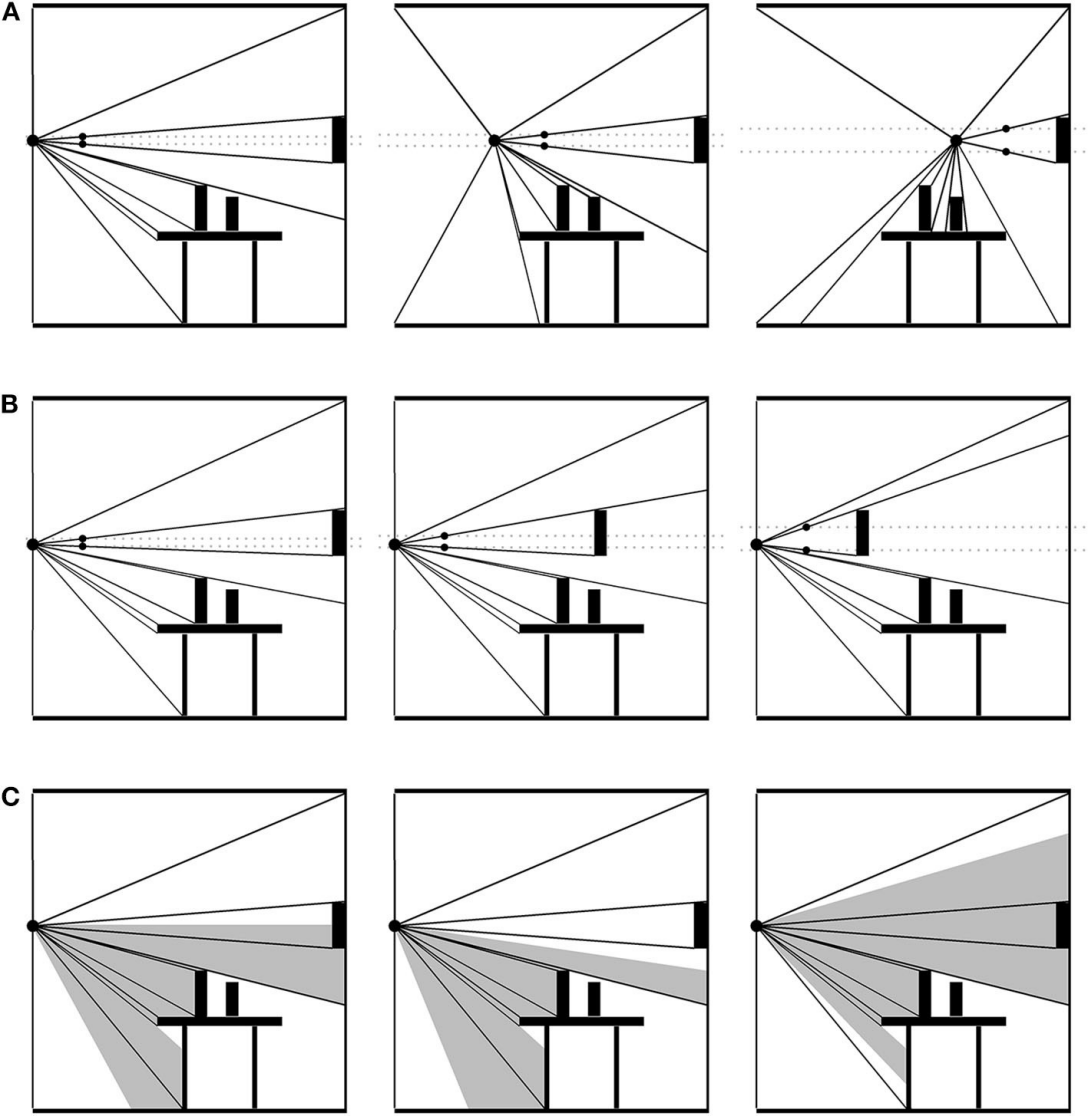
Optical flow field (OFF). A packed nesting of optical cones extends out from the point of observation to each surface. The OFF is structured by changes in surface details both large and small. **(A)** The point of observation (PO) moving across the scene. The gray, dashed lines illustrate the increasingly wide cone extending from the PO to the wall decoration. The black arrow points out a cone gradually disappearing as its surface disappears. The gray arrow points out cones accreting into the OFF as the PO approaches. Information about the environment's stationarity and the PO's non-stationarity is in the collective expansion and contraction of the field. **(B)** The point of observation remains stationary, but the wall decoration moves toward it. The change in the decoration's cone relative to the non-change in the rest of the field specifies that the object is moving and not the PO. **(C)** When the PO is an organism's line of sight, their particular embodiment will determine how much of the OFF is visible. Additional information about head movements, orientation, and location exists in the visible OFF.

An *optic field* consists of a packed nesting of reflected-light optical cones extending out from a single PO to all the edges and textures around it (see Figure 2). If the PO changes, or something in the environment changes in relation to the PO, the distribution of optical cones is transformed systematically. This change in distribution constitutes an *optic flow field* (OFF). Note that, as with catching a flyball, the PO need not be occupied by an eyeball or sensor on a creature or robot—it could just as well be a particle of dust. Nevertheless, an observer's perspective is limited by their embodied constitution, including their ocular apparatus, to only a portion of the OFF, the *visible optic flow field* (VOFF). The VOFF, embedded as it is in the OFF and defined as it is as a relation between the organism (its PO) and the objects in its environment, manifests a number of control laws. To adumbrate a few of them: moving forward expands the VOFF; moving backward contracts it; moving sideways translates it. The distribution of optical cones for an object moving toward the PO (at rest) will expand while the rest of the field remains the same; or, if the PO is also in motion, the object's optical cones will expand more rapidly, and it will occlude some of the others. Objects hidden behind others will introduce accrete or delete cones as it comes in and out of view. And so on (Gibson, 1979).

To get an idea of how control laws involving the optic flow field work, consider the hanging room experiment (Lee and Aronson, 1974). While standing still in a room without moving objects, the OFF will remain constant in all its relations. The VOFF will shift around with the viewer's saccades, but again, all relations in the distribution of cones will remain the same across saccadic transformations. This constancy is information about both the viewer and the environment's objects: namely, that neither is moving. Thus, if the “goal” is to remain standing still, the relationship between the OFF and the control structure embodied by the viewer is this: maintain constant OFF. If the entire OFF were to suddenly expand or contract, then this would imply that the viewer is moving forwards or backwards, respectively. Under normal circumstances, this is “specifying information” about the viewer's movement and the environment's lack of movement. Being dual with control, it also specifies *what to do* (Shaw and Turvey, 1981). If the OFF expands, move backward (to keep it constant), and move forward if it contracts (Lee and Lishman, 1975). The sliding room experiment subverts the usual relations, at least in part, by suspending the walls and ceiling of a room just above the floor, so that the room can move without moving the participant—at least, not physically. Moving the room *did* result in an “optic push,” explicable in terms of the foregoing control law. “Push” is used here because the response to the moving room is like that of a push, probably due to the mismatch between the moving room and a non-moving floor. Participants tripped backwards when the room moved toward them, and forwards when away. Children and inebriated adults fell over entirely. And again, this is without a physical push.

#### Tau (τ) Guidance

Tau is the ratio τ(*X, t*) = *X*(*t*)/Ẋ(*t*), which describes the time to close gap *X*, given the current rate of closure. In the case of control systems, the gap is between the current PO and a final (goal) PO. Rate $\overset{\cdot }{\tau }\left(X\left(t\right)\right)$ (alias, “tau dot”) can be used to guide controlled collisions and collision avoidance. Tau dot specifies whether the PO will make contact with a target, given the current rate of deceleration. Stopping *just at* or *before* colliding with an object requires stabilizing $\overset{\cdot }{\tau }$ to $\overset{\cdot }{\tau }\leq 1/2.\;$As with the OA and OFF, the gap *X* and its rate of closure Ẋ are constituted in the relationship between a PO and an object. Unlike OA and OFF, *X* and Ẋ cannot be detected. On the other hand, ρ(*t*) = 1/τ(*t*) = Ẋ(*t*)/*X*(*t*), a proportion of the rate of change to the current size of the gap, *can* be detected (Lee, 1998). In the case where final PO is also moving, synchronizing the closing of the two gaps means keeping their respective ρ in constant proportion, ρ(*X, t*) = λ_*X, Y*_ρ(*Y, t*), where *X* and *Y* are the two gaps, and λ_*X, Y*_ is a scaling factor setting the relative velocity curve. In the case of action guided by a gap closure with constant acceleration from rest, the rho ρ(*X,t*) = λ_*X,G*_ρ_*G*_(*G, t, T*_*G*_), where *T*_*G*_ is the time it takes to close the guiding gap *G*, and $\rho_{G}\left(G,t,T_{G}\right)=2t/\left(t^{2}-T_{G}^{2}\right)$. The equivalence means that ρ_*G*_ *prescribes* the value of λ_*x, G*_. In the case of a guiding gap *D* with constant deceleration, ρ_*D*_(*D, t, T*_*D*_) = 2/(*t*−*T*_*D*_) (Lee, 2018).

Moving beyond gaps between organism and environment, tau guidance provides a general scheme for control constituted by managing the opening and closing of gaps of all sorts, including pressure, angle, distance, and others (Lee et al., 2009). Information about gap closures can be found in relative rates of change ρ in *power* (its magnitude), whether found in ambient energy media, stimulation of sensory organs, or flows of electrochemical energy in a nervous or *phyto*nervous system (Calvo et al., 2017b; Lee, 2018). Similarly, with parameters of the efferent circuits, effectors, and action on the environment. None of this is to say that the organism *knows* anything about the powers, energies, or gaps anymore than a cat chasing and eating a mouse knows anything about its protein content. The control system generates gaps and ρ's, even as its gaps and ρ's are transformed by the environment. The control system *generates* information. And control laws exist where other parts of the control system are governed by it. As such, these information-generating ρ's amount to prescriptions for any other ρ's tied up in the interaction. Again, control is constituted in the coordination and synchronization of ρ's.

## Tau Guidance of Plant Nutation

Plant nutation is neither wholly endogenously nor wholly exogenously controlled, and it remains an open question whether nutation itself is due to internal oscillations, gravity-driven processes, or some combination of the two (Johnsson and Heathcote, 1973; Brown et al., 1990; Hejnowicz and Sievers, 1995; Johnsson et al., 1999; Charzewska and Zawadzki, 2006; Stolarz, 2009). The nutating tendril reaches out in all directions, taking stimulation at its receptors from the various structured energy fields in its environment. Stimulation at the sense organs is transformed into a variety of other energies, such as turgor pressure; flows of phytohormones and a number of other growth factors resulting in auxin redistribution and growth changes (Weisenseel and Meyer, 1997); and changes in electrical potential and ion transmission (Volkov, 2012). Much of this process takes place in dividing, meristematic embryological structures and courtesy of “rapid-long distance electrical and calcium signaling” (Choi et al., 2016) throughout the plant vascular system, has its effect in patterns of elongation and differentiation (Waddington, 1966) that underlie plant flexible, adaptive behavior (Calvo and Keijzer, 2011; Calvo et al., 2020).

Suppose that as the tendril makes its way around, nothing changes about the ambient energy media to which the plant has access. Stimulation of the sensory organs is constant. Perhaps the plant is surrounded by a climbable surface, but even so, the environment says nothing about where to go to get to it. The plant's nutation will go on, but undirected. On the other hand, there might be a tree branch. The plant will whip itself *toward* the branch, but *away* from the shade. The difference can be understood in terms of the ratio of red to far-red and blue to green light (Ballaré and Pierik, 2017)—the lower the ratio, the more shade from other plants, as they will have already absorbed the red and blue. Whatever the case, the plant's shade-avoidance movements will generate differences of stimulation at the plant's sensory organs, or gaps in stimulus power. And, when encountering these gaps, flows of phytohormone and other growth factors will generate auxin flows with yet further gaps, and so on, each process informed by its context of unequal distributions of power and their equalization.

### Control Laws Redux

Theory in ecological psychology is primarily concerned with interactive success. What are the necessary conditions for repeatable, reliable, successful encounters with the world? This is a matter that brings the entire ecosystem into focus—no organism can get by without reliable access to its environment. And no species comes into existence in a world where it has to do guess work and make inferences for more than a very small number of its activities. The layout of an environment and its lawfully structured energy media contain information, so the hypothesis goes, about *what to do*. As mentioned previously, control and lawful information are duals, they both define and entail one another (Shaw and Turvey, 1981).

The gap closures of tau theory may well be the most bountiful source of control laws in nature. The first gap any of us deal with in life is that between our own bodies and the floor or bed as we struggle against gravity. Indeed, the rate of closure of that gap is the ultimate prescriptive, guiding gap when we fall, as whatever action we take to reduce its damage must take place within it. Arguably, the optical flow field is a special case of tau-theoretic gap closure, as the changing distribution of optical cones can easily be conceived of as a packed nesting of opening and closing gaps. The chapman strategy involves the closing of not only the gap between ball and glove and ball and outfielder, but the closing in on a constant velocity, and so on.

## Conclusion

The preceding discussion suggests a reconsideration of what the challenges for robotics and artificial intelligence really are. Movement by growth, for instance, raises new questions. Artificial move-by-growing systems already exist (Mazzolai et al., 2010; Mazzolai et al., 2011), but can they explore their surrounds by growth? What about movement by exploration-guided growth? To ask such questions is to break with common intuitions centered narrowly around animals and their brains. It suggests that plants are like animals in being competent, agentic creatures capable of pursuing and realizing outcomes. And yet they differ from animals in being *brainless* and *morphologically plastic*, extending their surfaces outward, spatially and fractally. These facts suggest a need to rethink how we conceptualize agency.

The reader may be willing to admit that between organism or robot and environment, the control law is king, but once getting *inside* the thing, what then? A temptation exists to “scale up” with a hybrid between ecological and classical designs. For instance, Google bought Boston Dynamics. The former is known for world class artificial intelligence, and the latter state-of-the-art movement systems making heavy use of dynamics in their control structures. Perhaps the engineers at Google hoped to integrate them, as is the most logical conclusion if one assumes the body is a physical structure subject to the forces and flows of the real world, but the mind is some kind of computer. This partnership did not last long—why not? Our claim is that such endeavors are ill conceived in the first place. Organisms evolved their capabilities in dynamic, physical environments, impinged upon by a variety of forces, flows, and structured energy media. The organism need not guess about what to do most of the time, instead, it must *resonate* with what is already there (Raja, 2018, 2020; Fultot et al., 2019; Golonka and Wilson, 2019). The scheme is one of modulation of endogenous activity: *process informing*, rather than *information processing* (cf. Bickhard, 2015a,b; Fultot et al., 2019). The organism “tunes into” *lawful* information, and in doing so, *generates* information internally and further tunes its activities to *it*. In the case of tau theory, this means tau coupling. As the organism (robot) moves, it transforms stimulation at the sensors, generating gaps and taus/rhos. If the tau/rho in the environment prescribes tau/rho of a movement, then by picking up source power, stimulus power, then neural/phytoneural power, each tau/rho acts as a prescription for the next, an ensemble of coordinating degrees of freedom.

The foregoing provides a sampling of the richness available to a robotics and artificial intelligence for future research via ecological psychology and an emerging dialogue with neuroscience. Further models of “higher level” psychology exist within and around ecological psychology, such as direct learning (Jacobs and Michaels, 2007), resonance-based perceptual learning (Raja, 2019), and a wide array of others developed in interactivist theory (Bickhard and Richie, 1983; Bickhard, 1993, 2009, 2015a,b; Bickhard and Terveen, 1996).

## Author Contributions

PC conceived the original idea. PC and PF devised the project, the main conceptual ideas, and proof outline. PF worked out the technical details and wrote the manuscript in consultation with PC, LJ, and KA. All authors provided feedback and helped shape the manuscript.

## Conflict of Interest

The authors declare that the research was conducted in the absence of any commercial or financial relationships that could be construed as a potential conflict of interest.
